# Binder Free Hierarchical Mesoporous Carbon Foam for High Performance Lithium Ion Battery

**DOI:** 10.1038/s41598-017-01638-y

**Published:** 2017-05-03

**Authors:** Zhengping Zhou, Hua Zhang, Yan Zhou, Hui Qiao, Ashim Gurung, Roya Naderi, Hytham Elbohy, Alevtina L. Smirnova, Huitian Lu, Shuiliang Chen, Qiquan Qiao

**Affiliations:** 1 Center for Advanced Photovoltaics, Department of Electrical Engineering and Computer Sciences, South Dakota State University, Brookings, SD 57007 USA; 2 Department of Chemistry and Chemical Engineering, Jiangxi Normal University, Nanchang, 330022 China; 3 School of Textiles and Clothing, Key Laboratory of Eco-Textiles, Ministry of Education, Jiangnan University 1800 Lihu Avenue, Wuxi, 214122 China; 4 Department of Chemistry and Applied Biological Sciences, South Dakota School of Mines and Technology, Rapid City, SD 57701 USA; 5 Construction and Operations Management, South Dakota State University, Brookings, SD 57007 USA

## Abstract

A hierarchical mesoporous carbon foam (ECF) with an interconnected micro-/mesoporous architecture was prepared and used as a binder-free, low-cost, high-performance anode for lithium ion batteries. Due to its high specific surface area (980.6 m^2^/g), high porosity (99.6%), light weight (5 mg/cm^3^) and narrow pore size distribution (~2 to 5 nm), the ECF anode exhibited a high reversible specific capacity of 455 mAh/g. Experimental results also demonstrated that the anode thickness significantly influence the specific capacity of the battery. Meanwhile, the ECF anode retained a high rate performance and an excellent cycling performance approaching 100% of its initial capacity over 300 cycles at 0.1 A/g. In addition, no binders, carbon additives or current collectors are added to the ECF based cells that will increase the total weight of devices. The high electrochemical performance was mainly attributed to the combined favorable hierarchical structures which can facilitate the Li^+^ accessibility and also enable the fast diffusion of electron into the electrode during the charge and discharge process. The synthesis process used to make this elastic carbon foam is readily scalable to industrial applications in energy storage devices such as li-ion battery and supercapacitor.

## Introduction

A rechargeable lithium-ion battery (LIB), typically consisting of an intercalated lithium compound cathode, an anode, and an electrolyte, stores and releases energy by simultaneous extraction and insertion of lithium ions and electrons^1, 2^. LIBs power most of today’s portable electronic devices and electric/hybrid vehicles due to their superior stored volumetric and gravimetric energy densities^3, 4^. However, the increasing demand of high power density, low cost, fast charge/discharge rates and long life cycles together with light weight and flexibility requirements are driving further improvements in the LIB technology^5^. Therefore, tremendous research efforts are needed to facilitate the development of current LIBs by developing new electrode materials and electrolytes, innovative device design and maximizing active material content in devices^6–8^.

Carbon based materials including carbon nanotubes (CNTs), carbon nanofibers (CNFs), carbon foam (CF) and graphene nanosheets have attracted considerable attention in recent years by virtue of their high specific surface area, superior electrical conductivity and excellent thermal stability^9–12^. Among these, CF is a 3D interconnected porous carbon material and has been studied as a highly electrically conductive network, robust electrical and mechanical support with sufficient space for a high energy density metal oxides loading^4–6, 13–15^. Only a few seminal work has investigated the low-density mesoporous CF as anodes of LIBs^16–18^. For example, Elena Rodríguez and co-workers have developed a boron-doped CFs by the co-pyrolysis of a coal and two boron sources^16^. They documented that the boron oxide-based CFs have a larger reversible lithium ion storage capacities of 310 mAh/g. However, the extremely high temperature of 2800 °C for pyrolysis may results in a high cost of manufacturing in industrial scale^16^. Additionally, increasing consumer and industrial demand also requires compact, low density, or flexible/or stretchable battery devices together with high energy and power densities.

As we have reported previously^19^, an elastic carbon foam (ECF) was synthesized by a simple two-step process. The ECF possess unique properties including excellent electrical conductivity, thermal properties and high specific surface area, ultrahigh porosity (99.6%) and low density (5 mg/cm^3^). Herein, in this study, the ECF with a high specific surface area of 980.6 m^2^/g and narrow pore size distribution of ~2–5 nm was directly prepared as anode materials without any binder and conductive additives for a high-performance LIBs. Such multifunctional foam with 3D interconnected network architecture can facilitate the lithium ion accessibility and electron transport. The integration of ECF in advanced electrochemical electrical energy storage devices, such as batteries, supercapacitors, and solar cells, may provide a solution to meet the consumer and industrial demands for lightweight, flexible and long-lasting energy applications.

## Experimental

### Preparation of elastic carbon foam (ECF)

Melamine-formaldehyde foam (MF) was obtained from SINOYQX (Sichuan province, China) and used as received. The ECF was fabricated from MF through a simple two-step pyrolysis process: oxidizing at 300 °C in air and then carbonizing at 1000 °C in N_2_ using the following routes. A piece of MF (90 × 50 × 30 mm) was first placed at porcelain boat in a quartz tube (diameter Φ = 80 mm, length = 1000 mm). The quartz tube was heated to 300 °C with a heating rate of 1 °C/min in air and annealed at the same temperature for 0.5 h to stabilize the MF; then heated to 1000 °C with a heating rate of 10 °C/min in N_2_ atmosphere, and annealed for 0.5 h at 1000 °C. After the annealing procedure, the sample was then cooled to room temperature. The whole process of preparation of ECF is unique and simple and the ECF can be reproduced.

### Sample characterization

The morphology and microstructure of the as-prepared ECF were observed by a Hitachi S-4300N scanning electron microscope (SEM) and a JEM-2010 transmission electron microscope (TEM), respectively. Raman spectroscopy (Lab Ram HR Jobin Yvon) and X-ray diffraction (XRD, Siemens D5000) were also employed to analyse the carbon structures of the ECF. The Brunauer-Emmett-Teller (BET) specific surface area and porosity of ECF were characterized using nitrogen sorption isotherm (Micromeritics Instrument Corp.).

### Electrochemical measurements

The electrochemical performance of the ECF was examined using CR2032 coin cells. The ECF was cut into a suitable size (~1.3 cm^2^) and directly used as a working electrode. Note that neither conventional conductive such as carbon black nor binder such as PVDF were used for the anode and the ECF was directly placed onto positive cell case. A pure lithium chip (99.9%, MTI Corp.) worked as both the counter and reference electrodes. The liquid electrolyte used was 1M LiPF_6_ in a mixture organic solvent of ethylene carbonate (EC)/diethyl carbonate (DC)/dimethyl carbonate (DMC) (1:1:1 in volume, MTI Corp.). The separator used was a 25 µm Trilayer polypropylene- polyethyerlene-polypropylene membrane (Celgard). The cells were assembled in an argon-filled glovebox. The galvanostatic charge-discharge tests were carried out on a LAND CT2001A battery tester system at room temperature in a potential window of 0.005–3.0 V (vs. Li/Li+) by varying a current density of 0.04–1.0 A/g. The electrochemical impedance spectroscopy (EIS) tests were conducted on amplitude of 5 mV over the frequency in the range of 100 kHz–1 Hz.

## Results and Discussions

### Morphology and structure

**Figure 1:**
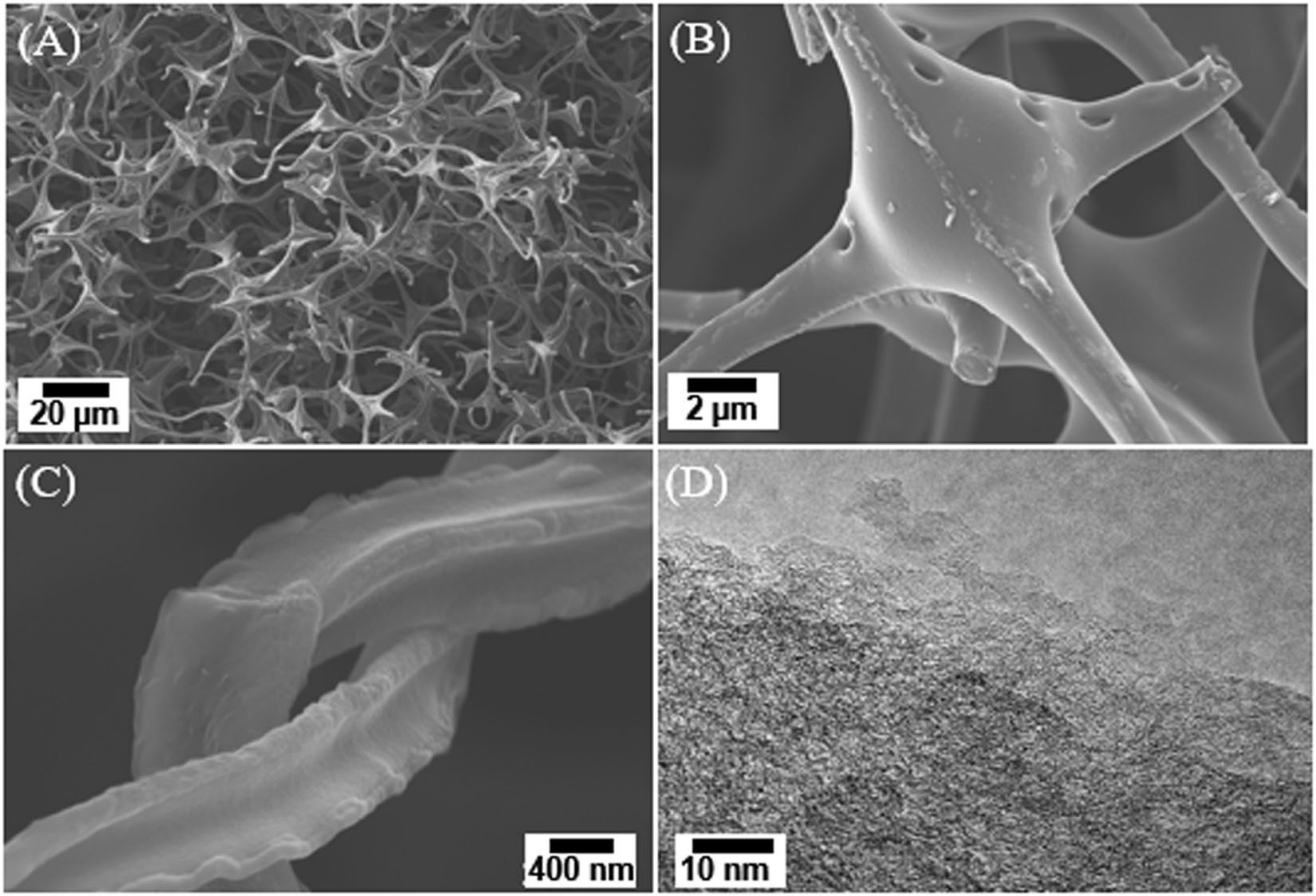
Top view SEM images of ECF at (**A**) low and (**B**,**C**) high magnification, respectively; (**D**) high-resolution TEM image of the thin edge of ECF.

The oxidation step of melamine-formaldehyde foam (MF) at 300 °C in air leads to the formation of carbonyl group, which is needed to stabilize the MF prior to carbonization. The oxidized MF was then carbonized at 1000 °C under N_2_ flow. The effect of pyrolysis temperature (700, 800, 900, 1000, and 1800 °C) on the morphology and microstructure of ECF has been investigated in our previous report^19^. In this study, we focused on the 1000 °C-ECF electrode for use in LIBs. The overall morphology and structure of the ECF were well maintained despite a significant shrinkage of the foam during carbonization. Figure 1(A) shows the representative scanning electron microscopy (SEM) image of the pure ECF. Figure 1(B,C) show high-resolution SEM images. The ECF possesses 3D interconnected carbon network architecture and concave triangle fiber shape. The diameter of the ECF fibers is approximately 1–2 μm. Few macro- and meso-pores on the fiber surface were formed in the oxidative stabilization and carbonization processes. The 3D interconnected carbon network with macro- and meso-pores could serve as an electrolyte volume reservoir for facilitating ion diffusion to the active material^6^. High-resolution transmission electron microscope (HR-TEM) image (Fig. 1D) shows the microstructure of the ECF, indicating the carbonization process has generated a 3D distribution of mesopores. These pores have small size ranging from ~2 to 5 nm. In addition, as described previously^19^, the ECF has advantages of excellent elasticity and ultra-low density of ~5 mg/cm^3^. The electrical conductivity of the ECF was about 6.8 × 10^−2^ S/cm measured by the four-point probe method^19^. Hence, the ECF is favorable for serving as a porous electrode with mechanical support and an electrically conductive network. The 3D interconnected porous ECF with unique macroporous carbon skeleton could effectively enhance the lithium ion storage capacity. The reason is that the macro- and meso-pores of ECF could provide fast transportation paths for lithium ions and the mesopores further enhance the active sites for the storage of lithium ions^13^.

**Figure 2:**
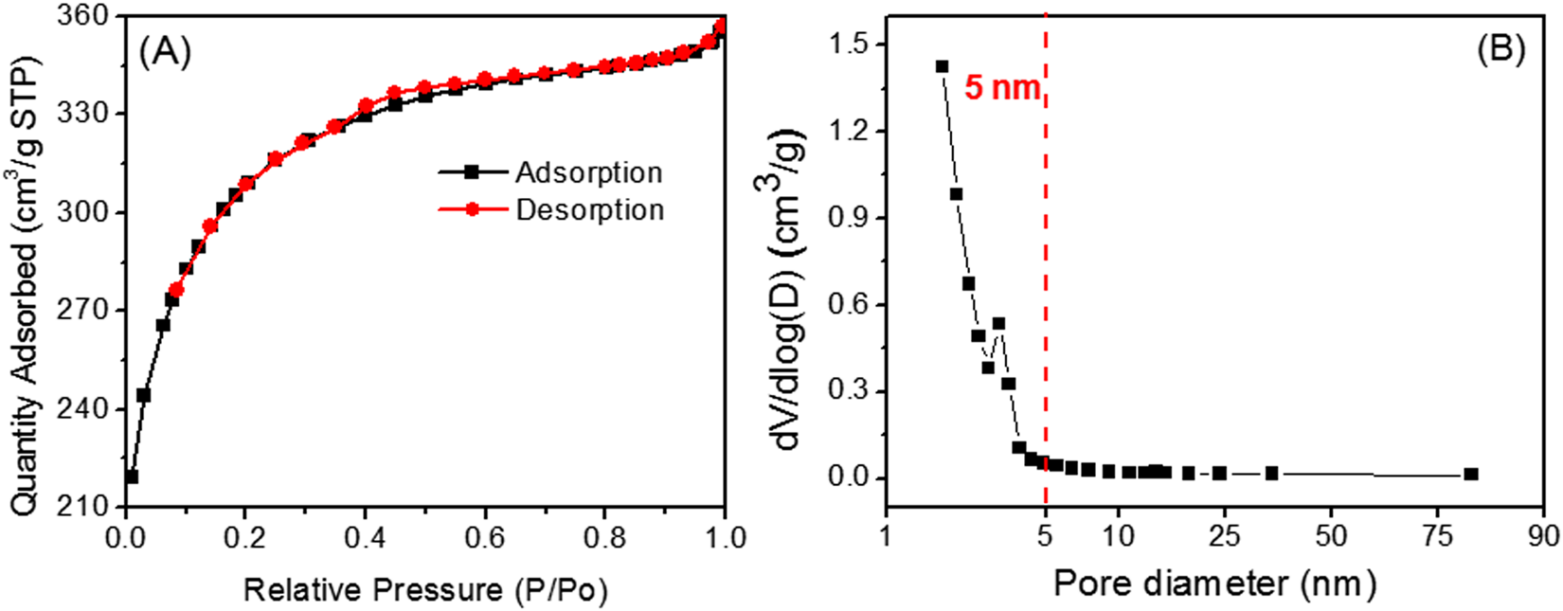
(**A**) N_2_ absorption and desorption isotherms linear plot and (**B**) pore size distribution of the ECF materials.

The specific surface area and pore size of the ECF were measured by the nitrogen (N_2_) absorption/desorption isotherms. As shown in Fig. 2(A), the N_2_ isotherm revealed a type IV isotherm characteristic and a hysteresis loop at relative pressure (P/P_0_) of 0.3–0.6, suggesting the presence of mesopores and pore condensation from the interconnected pore system of the ECF^4, 20^. The specific surface area was calculated in the relative pressure range from 0.06 to 0.3. The ECF exhibited a high BET specific surface area of 980.6 m^2^/g. Such high surface area of the ECF is mainly attributed to the release of nitrogen atoms as N_2_ and NH_3_ gases during the carbonization process which results in the formation of mesopores^19^. The higher specific surface area of the ECF could provide more electrochemical active sites to enhance lithium ion storage and adsorption capability. Additionally, the higher surface area of carbon foam can provide a more effective interface with electrolyte material and porous bulk by giving rise to a higher compatibility with other conductive additives such as transition metal oxides. The pore size distribution plot is shown in Fig. 2(B). The analysis of the N_2_ adsorption data further demonstrated the presence of mesopores ranging from ~2 to 5 nm as well as micropores less than ~2 nm with a total pore volume of 0.55 cm^3^/g. As calculated, the micropore and mesopore (>2 nm) volumes are 0.25 and 0.30 cm^3^/g, respectively. The distribution of pore sizes corresponds to the observation from TEM images (Fig. 1D).

**Figure 3:**
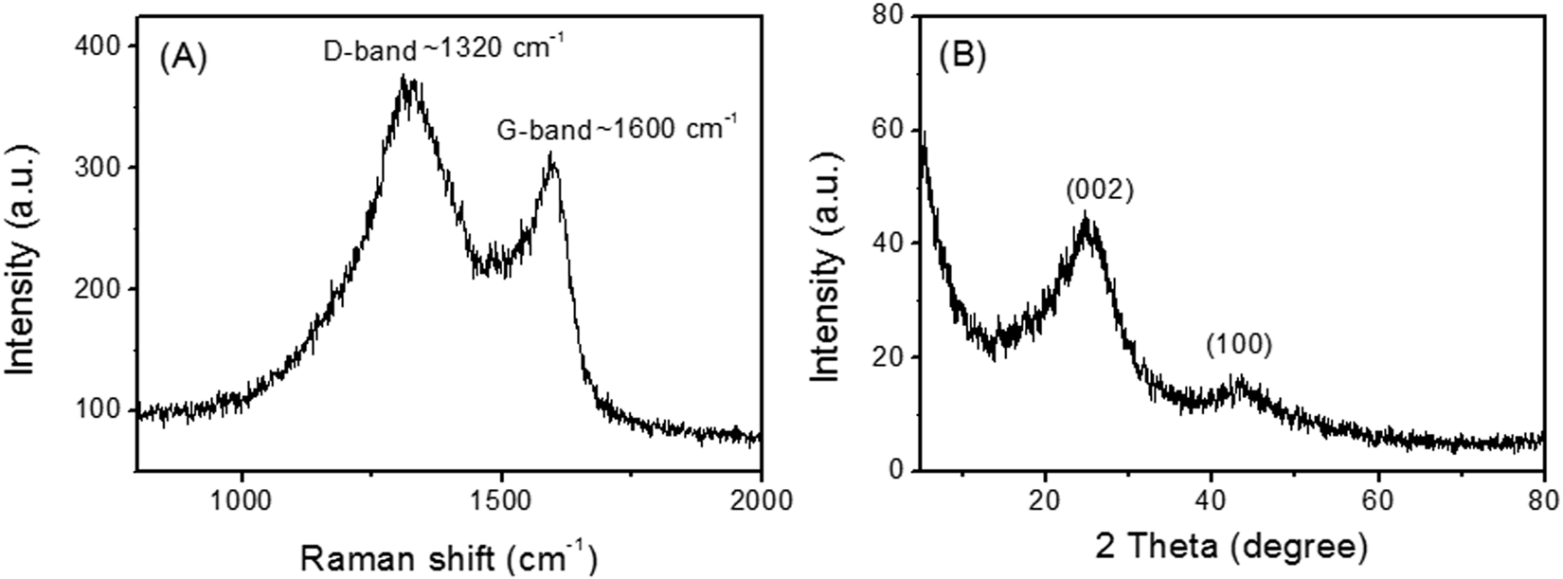
Raman spectroscopy (**A**) and XRD characteristic (**B**) of elastic carbon foam.

Raman spectroscopy and X-ray diffraction (XRD) are two techniques that can be used to characterize carbon allotropes. Although all the carbon materials are formed upon C-C bonds, the orientation of C-C bonds alters from one structure to the other. Since graphite is well known Li ion battery electrode material, the Raman and XRD characterizations of the carbon foam were analysed using graphite as the reference. Raman characteristic peaks of sp^2^ hybridized C=C bonds in graphite known as D1, D2 and G band, normally occur at 1350,1362 and 1580 cm^−1^, respectively. D-bands correspond to the surficial defects or edge planes while G-band represents the interior of the perfect crystalline structure in which G-bands normally have higher intensity of Raman scattering than that of D-bands^21^. As shown in Fig. 3(A), the Raman peaks of ECF at ~1320 and ~1600 cm^−1^ indicate the shifted D and G bands, respectively. The higher surface area of carbon foam compared to graphite attributed to the presence of more dangling bonds and more intrinsically light scattering behaviour of the edges. The peak shift from 1350–1360 cm^−1^ in graphite to 1320 cm^−1^ in carbon foam illustrates the mechanical or thermal effects on lattice as a product of foaming process. However, the D-band in carbon foam reveals properties closer to single layered graphene C=C bond rather than the graphite structure^22^. Raman spectroscopy results demonstrated roughly similar band structures of ECF and graphite.

Graphitic crystalline structures oriented in known lattice directions are reported by many others such as C (002), C (100), C (110) and C (004) associated to XRD peaks of 2Ɵ = 26.5°, 44.3°, 77° and 53°, respectively^21, 22^. Likely, the most intense peak indicates that the highest crystalline order occurs at C (002) plane where 2Ɵ = 26.5°. In this study, the purity analysis of ECF and its crystallinity resemblance to the graphite were investigated^23^. As shown in Fig. 3(B), the XRD characteristic peaks of ECF were centred at 2Ɵ = 25° and 43° corresponding to C (002) and C (100) crystalline plane orientations, respectively^21, 24^. The interplanar spacing of the lattices were calculated using Bragg’s law and found to be 3.55 A° for 2Ɵ = 25° and 2.1 A° for 2Ɵ = 43°. Compared to conventional C (002) d-spacing of graphite at 3.36 A°, there was a 0.19 A° transition in interplanar spacing of this lattice while compared to C (100) d-spacing of graphite at 2.04 A°, there was a 0.06 A° transition. The peak at 2Ɵ = 25° is sharper than that at 2Ɵ = 43° following the same aforementioned property of graphite. This is attributed to the presence of the single layered graphitic structure in carbon foam as discussed in Raman spectroscopy analysis. The broader peak width in ECF is affected by the single layered graphitic structure existing mostly at the edges, which lowers the crystallinity and relative electronic properties at such regions. However, the higher chemical reactivity of the edges in ECF can engage more interfacial reactions with the electrolyte which facilitates the ionic properties of battery internal reactions.

### Electrochemical properties

**Figure 4:**
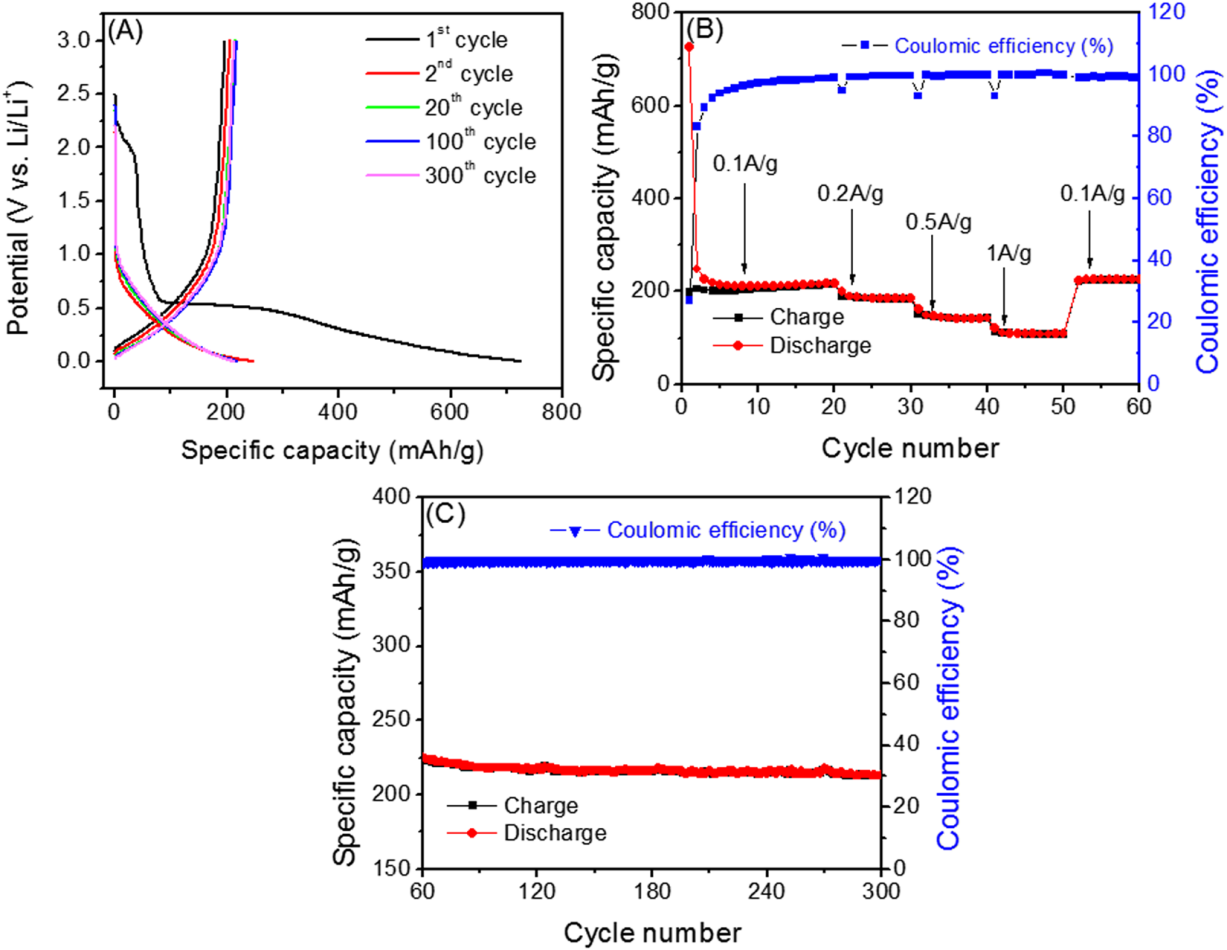
(**A**) Galvanostatic charge-discharge tests at a current density of 0.1 A/g (0.5 C); (**B**) Rate capability of the ECF electrode with a thickness of 13.7 mm at different current densities; (**C**) Subsequent cycling tests at 0.1 A/g (0.5 C) from 60^th^ to 300^th^ cycle.

The electrochemical performance of interconnected porous ECF based cells was investigated by galvanostatic charge-discharge tests. Figure 4(A) shows the discharge-charge voltage profiles of the electrode made of the ECF at the 1^st^, 2^nd^, 20^th^, 100^th^, and 300^th^ cycles with a current rate of 0.1 A/g (0.5 C) in the potential window of 0.005−3.0 V (vs. Li/Li^+^). The initial charge curve presents a short potential plateau at around 2.25–2.0 V and a long potential plateau starting from ~0.5 V. The formation of the two plateaus is due to the irreversible electrolyte decomposition and the formation of SEI films on the surface of ECF. The specific capacity was calculated based on the weight of ECF. The ECF can attain a high initial discharge capacity of 725 mAh/g and charge capacity of 199 mAh/g, respectively. Despite the discharge capacity loss after the irreversible initial cycles, the reversible capacity of ECF decreased from ~206 mAh/g at the 2^nd^ cycle to ~201 mAh/g at the 5^th^ cycle, but then increased to ~215 mAh/g at the 20^th^ cycle. The capacity reached maximum of ~225 mAh/g at the 56^th^ cycle and maintained at 213 mAh/g up to 300 cycles (Fig. 4C).

Furthermore, the rate capability of the ECF was measured at various current densities of 0.1 (0.5 C), 0.2 (1.1 C), 0.5 (3.8 C) and 1 A/g (10 C) as shown in Fig. 4(B). The reversible capacities of the ECF at various current rates of 1.1, 3.8 and 10 C are 184, 142 and 108 mAh/g, respectively. Note that when the current density was reversed to 0.1 A/g (0.5 C) starting from 50^th^ cycle, a high reversible capacity of ~220 mAh/g can be maintained. The initial coulombic efficiency of the ECF is 27.4% due to the irreversible capacity loss ~500 mAh/g, but then significantly increased to 99.1% in 20 cycles. The 2^nd^ cycle charge and discharge capacities of 206 and 248 mAh/g respectively showed a significant enhanced coulombic efficiency of 83.1%,. The coulombic efficiency of the ECF maintained ~100% from 60^th^ to 300^th^ cycles as shown in Fig. 4(C).

**Figure 5:**
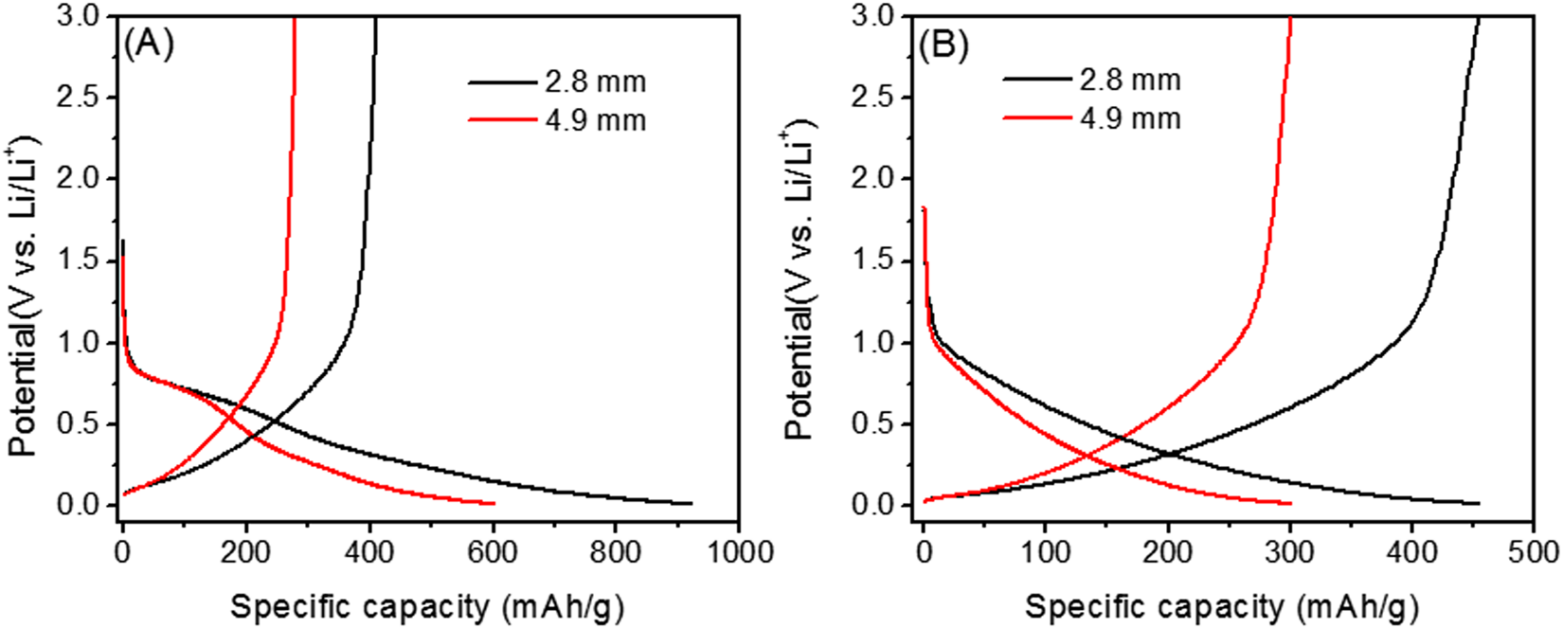
Galvanostatic charge-discharge tests of the ECF with different thicknesses at a current density of 0.04 A/g at 1^st^ (**A**) and 100^th^ cycle (**B**).

The dependence of anode thickness on the charge-discharge capacities, rate capability and cyclability have been comparatively investigated under the same condition. In this experiment, the ECF with the density of ~ 5 mg/cm^3^ was cut into two thin films with thicknesses of 2.8 mm (~1.8 mg) and 4.9 mm (~3.2 mg), respectively. These ECF electrodes were then assembled into Li-ion cells under the above same experimental conditions. Figure 5(A) and (B) show the galvanostatic charge-discharge curves of ECF electrodes at 1^st^ and 100^th^ cycles, respectively. Similarly, the potential of the cells quickly decreased from the open circuit potential to ~1.0 V. Different with the previous ECF electrode (thickness: 13.7 mm), the two thinner ECF electrodes only present one potential plateau starting from ~1.0 V. The charge and discharge capacities and coulomic efficiency at 1^st^ and 100^th^ cycles were extracted from Fig. 5(A) and (B) and shown in Table 1. Clearly, both charge and discharge capacities of the cells are strongly dependent on electrode thickness. The 1^st^ discharge and charge capacities of the 2.8-mm ECF electrode are 921 and 410 mAh/g, respectively, which are higher than that of conventional graphite anodes of 372 mAh/g^25^ and the reported value of 310 mAh/g of graphitized boron-doped carbon foams^16^. When the ECF electrode thickness increased to 4.9 mm, the discharge and charge capacities significantly decreased to 602 and 279 mAh/g, respectively. This demonstrated that the thinner ECF electrode can deliver higher charge and discharge capacities than that of the thicker electrode which will give a lower irreversible capacity loss due to less exposed outer surface area at the electrode/electrolyte interface^26^. In addition, the Coulomic efficiency of the first cycle was about 45% for both 2.8- and 4.9-mm electrodes, which is much higher than that of the 13.7 mm electrode of 27.4%. In the ECF nanostructure, the thinner electrode provides a much stronger interaction with the surrounding Li ions than that of the thick electrodes due to their larger exposed surface area with respect to the total mass. Some reported the thinner anode yields superior performance due to faster kinetics and less disruption of the electrode morphology^26^, lower electronic resistance of the electrode, and faster transport of Li ions in the electrolyte to the electrode surface and diffusion of Li ions within the bulk electrode^27^.

**Table 1:** Comparison of electrode, charge and discharge capacities, and coulomic efficiency of the ECF electrode at the 1^st^ and 100^th^ cycle.

ECF electrode thickness (mm)	Charge capacity (mAh/g) 1^st^ & 100^th^	Discharge capacity (mAh/g) 1^st^ & 100^th^	Coulomic efficiency (%) 1^st^ & 100^th^
2.8	410 & 454	921 & 455	44.5 & 99.8
4.9	279 & 301	602 & 300	46.3 & 100.3
13.7	199 & 218	725 & 219	27.4 & 99.5

**Figure 6:**
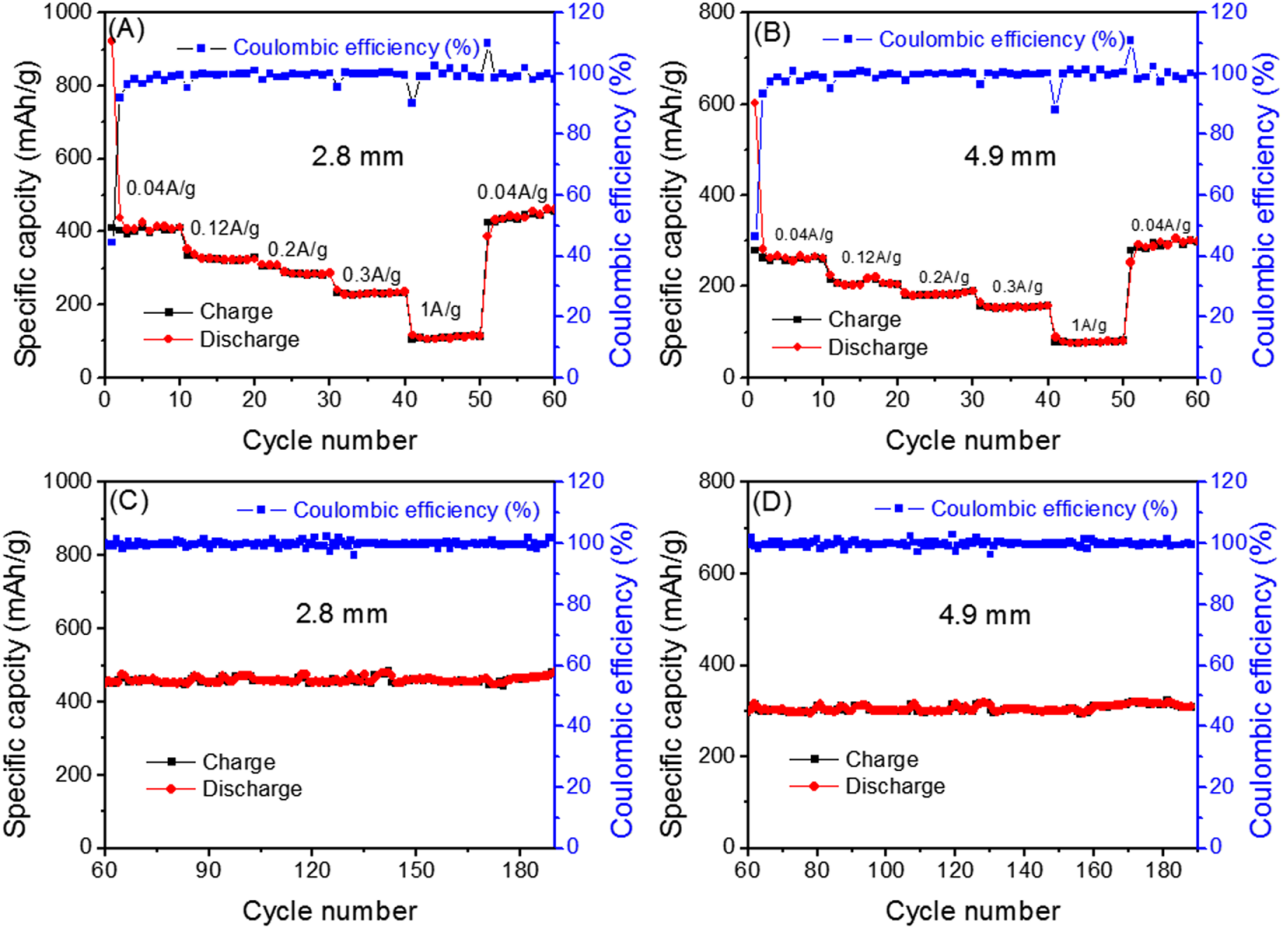
Rate capability of the ECF with a thickness of 2.8 mm (**A**) and 4.9 mm (**B**) varying current densities from 0.04 to 1 A/g; Subsequent cycling tests of the ECF electrodes with thickness of 2.8 mm (**C**) and 4.9 mm (**D**) at 0.04 A/g from 60^th^ to 190^th^ cycle.

The rate capabilities of the 2.8- and 4.9-mm ECF electrodes were also investigated at current densities of 0.04, 0.12, 0.2, 0.3 and 1 A/g between 0.005–3.0 V (vs. Li/Li+), as presented in Fig. 6(A) and (B). The charge and discharge capacities of the 2.8- and 4.9-mm ECF electrodes decreased as the current density increased. For example, the 2.8-mm electrode exhibited the highest specific capacity at all current densities, which are 455, 352, 307, 234 and 115 mAh/g at current densities of 0.04, 0.12, 0.2, 0.3 and 1 A/g, respectively. In addition, both 2.8- and 4.9-mm ECF electrodes demonstrated an excellent coulombic efficiency of ~100% at high current rate charge-discharge, indicating a high capability of sustaining high current density. It must be noticed that the reversible capacities for the 2.8- and 4.9-mm ECF electrodes continuously increased from 426 to 455 mAh/g and 279 to 300 mAh/g starting from 51^st^ to 60^th^ cycles, respectively. The remarkable increase of capacity is corresponded to the absorbing behaviour of Li ions on the ECF nanostructure surface, in good agreement with several earlier works^9, 28^.

The highest capacities of 455 and 300 mAh/g for the 2.8- and 4.9-mm ECF electrodes can still be maintained to 190 cycles as shown in Fig. 6(C) and (D). After the first cycle, the Columbic efficiency of the 2.8- and 4.9-mm ECF electrodes jumped to ~100%, indicating excellent cycle stability (this low initial irreversibility is due to SEI film formation). Therefore, it can be concluded that the battery using thinner ECF electrodes exhibited much higher specific capacity with respect to the thicker electrodes.

**Figure 7:**
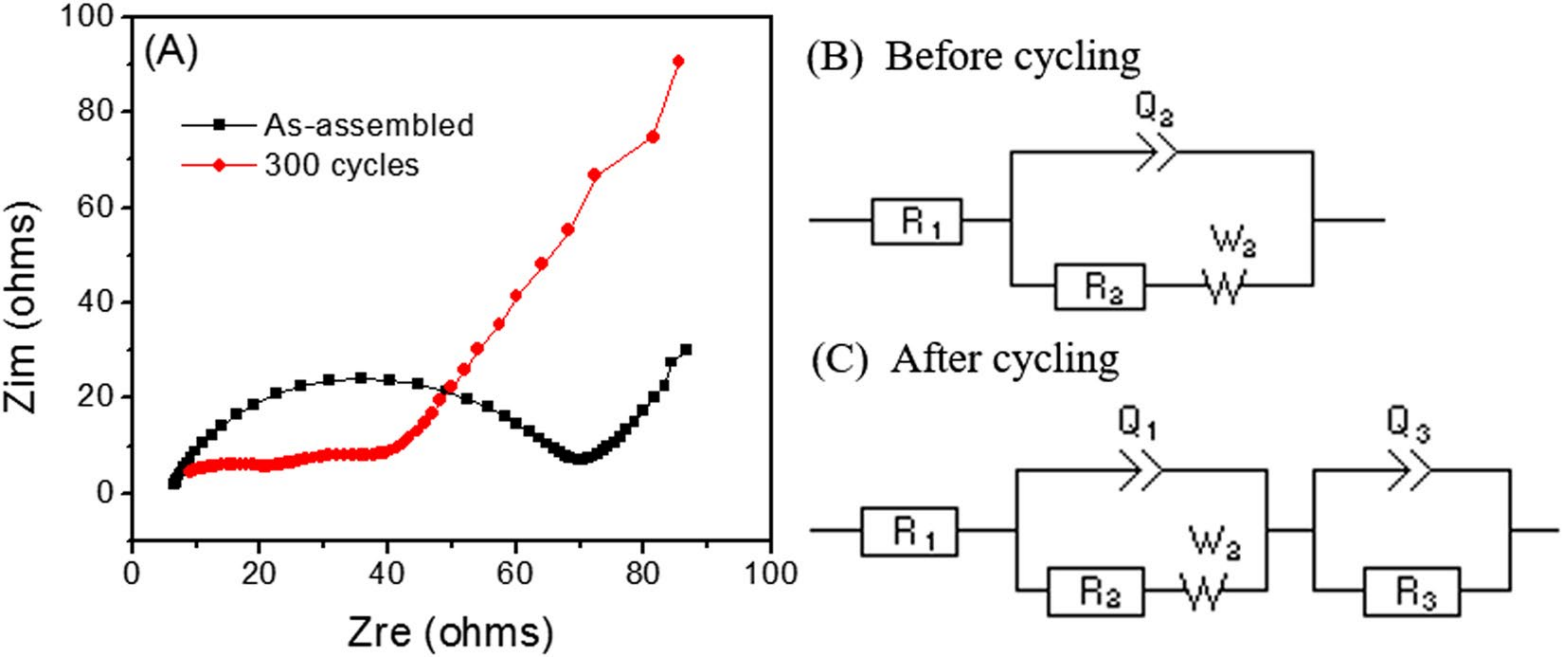
(**A**) Nyquist plots of the ECF under different cycling conditions: as-assembled cycle and after 300 cycles. Equivalent circuits for fitting Nyquist plots (**B**) before and (**C**) after cycling. R_1_: the internal series resistance of the cell; R_2_: the charge-transfer resistance; and R_3_: the SEI film resistance.

In order to understand the effect of charge-discharge cycles on the electrochemical performance of LIBs, electrochemical impedance spectroscopy was used to examine one of the ECF electrodes (i.e. 13.7 mm) during the as-assembled and after 300 cycles. Figure 7(A) presents the Nyquist plots of the ECF electrode with a thickness of 13.7 mm base cell under different cycling conditions: as-assembled and after 300 cycles, respectively. The Nyquist plots possess a semicircle in the high-medium frequency region. An equivalent circuit model (Fig. 7B) was used for fitting the plots from the ECF cell before cycling, where R_1_ is the internal series resistance of the cell, R_2_ is the charge-transfer resistance, Q_2_ is the constant phase element of the electrode/electrolyte interface, and W_2_ is the Warburg resistance corresponding to the lithium diffusion process^14^. After 300 cycles, another equivalent circuit (Fig. 7C) was applied to fit the ECF impedance spectra. R_3_ is the resistance of solid electrolyte interface (SEI) and Q_3_ represents constant phase element of SEI^14^. The Nyquist plot of the ECF electrode after 300 cycles has two small semicircles. The first semicircle is attributed to the combination of SEI and charge-transfer resistance, while the second semicircle is mainly caused by the bulk resistance which is effected by both the electronic conductivity of active materials and the electrolyte resistance^28, 29^.

The resistance of the ECF cell before cycling and after 300 cycles were extracted by fitting the Nyquist plots and shown in Table 2. The values of R_1_ and R_2_ of the ECF cell after 300 cycles are 3.25 and 22.20 Ω compared to 7.15 and 52.68 Ω from the ECF cell before cycling. A smaller R1 indicates the electrode/electrolyte resistance of the ECF cell decreased with the increase of the cycle number, which demonstrated a well-sustained electronic contact of the ECF with the electrolyte. On the other hand, a significant decrease of R_2_ of the ECF cell from the initial to 300^th^ cycle, indicating the formation of SEI could enhance the solid-state lithium ion diffusion rate^15^.

**Table 2:** Comparison of fitted impedance parameters of the ECF electrode (thickness: 13.7 mm) before cycling and after 300 cycles.

ECF anode	R_1_ (Ω)	R_2_ (Ω)	R_3_ (Ω)
As-assembled	7.15	52.68	NA
300 cycles	3.25	22.20	8.94

**Figure 8:**
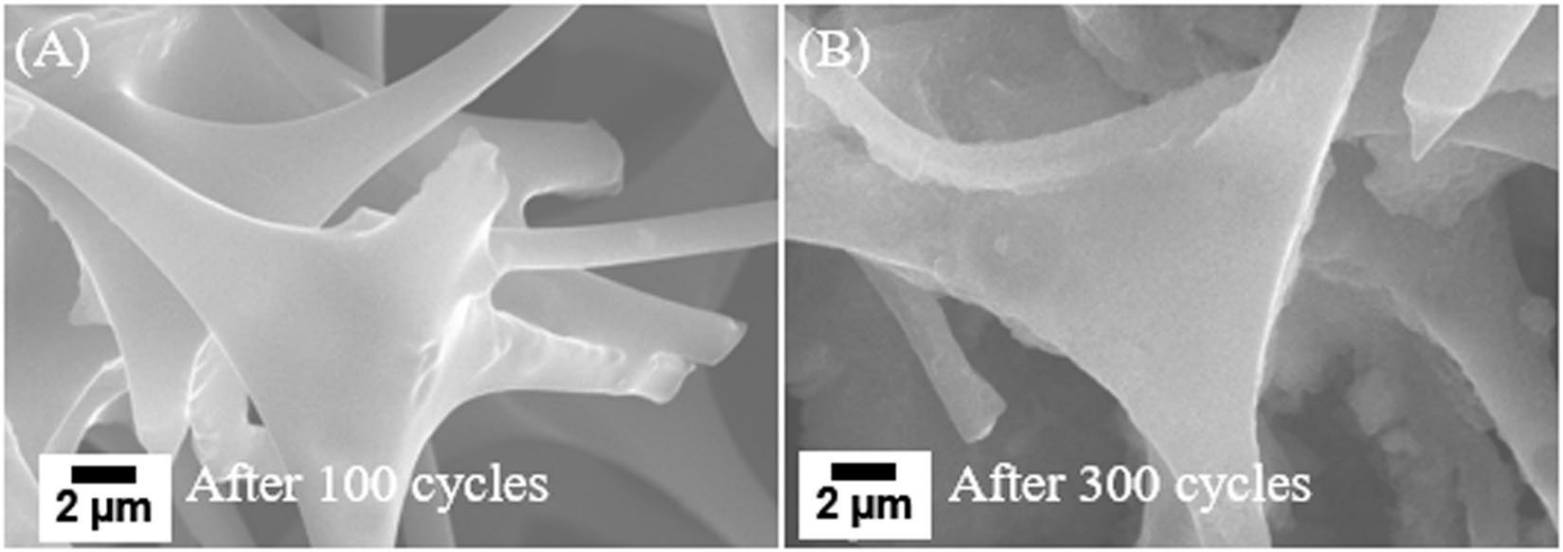
SEM images of the ECF electrode after (**A**) 100 and (**B**) 300 cycles at 0.1 A/g, respectively.

Post-cycling SEM characterization was utilized to investigate the effects of the long-term charge-discharge cycling on the morphology and microstructure of the ECF electrode. As shown in Figs 8(A) and S2, the ECF electrode with a thickness of 13.7 mm (~9.0 mg) after 100 charge-discharge cycles at 0.1 A/g still inherits the interconnected carbon network architecture and macropores as well as the original shape of the ECF fibers, indicating a very high morphological and structural stability of the ECF electrode. Figures 8(B) and S3 shows the SEM image of the cycled ECF electrode after 300 charge-discharge cycles at 0.1 A/g. A little bit expansion (~5% in volume) on the fiber diameter occurred during such the long-term cycling process due to the Li ion intercalation or absorption on the ECF fibers. The reaction products were accumulated on the surface of the ECF electrode and the pores were disappeared due because of the incomplete reversible electrochemical process (Figs  S1 and 3). However, the original morphology of the ECF electrode is still maintained. The high specific surface area, narrow mesopore size distribution and high electrical conductivity the ECF electrode can adsorb and store Li ions more easily and relieve the interlayer stress by volumetric expansion^28^.

## Conclusions

In summary, a novel 3D hierarchical interconnected mesoporous ECF was synthesized by a simple two-step method including oxidation and carbonization of Melamine-formaldehyde foam (MF). A high specific surface area (980.6 m^2^/g) and narrow mesopore size distribution (~2–5 nm) guaranteed the binder-free ECF anodes to have a high reversible capacity of 455 mAh/g. More importantly, the coulombic efficiency of the ECF remained almost 100% for 300 cycles at 0.5 C. Such excellent performance obtained together with a two-step, facile and cost-effective production process demonstrated this ECF could be a promising anode material for advanced energy/power electrochemical electrical energy storage devices. Furthermore, the ECF with such porous carbon structure can be used as support matrix for high capacity alloy anode materials such as Si, Sn for suppression of volumetric expansion during cycling.

## Electronic supplementary material

Binder Free Hierarchical Mesoporous Carbon Foam for High Performance Lithium Ion Battery
