# Supplementary material for: Binder Free Hierarchical Mesoporous Carbon Foam for High Performance Lithium Ion Battery
Source: Sci Rep. 2017 May 3;7:1440. doi: 10.1038/s41598-017-01638-y (PMC5431229; doi:10.1038/s41598-017-01638-y)
Supplement: Supplementary file 1 — Binder Free Hierarchical Mesoporous Carbon Foam for High Performance Lithium Ion Battery [file 41598_2017_1638_MOESM1_ESM.pdf]

Supporting Information:

Binder Free Hierarchical Mesoporous Carbon Foam for High Performance Lithium Ion Battery

Zhengping Zhou[a, †], Hua Zhang[b, †], Yan Zhou[b], Hui Qiao*[c], Ashim Gurung[a], Roya Naderi [a], Hytham Elbohy[a], Alevtina L. Smirnova[d], Huitian Lu[e], Shuiliang Chen*[b], and Qiquan Qiao*[a]

[a]*Center for Advanced Photovoltaics, Department of Electrical Engineering and Computer Sciences, South Dakota State University, Brookings, SD, 57007, USA.* Qiquan.Qiao@sdstate.edu

[b]*Department of Chemistry and Chemical Engineering, Jiangxi Normal University, Nanchang, 330022, China.* slchenjxnu@jxnu.edu.cn

[c]*School of Textiles & Clothing, Key Laboratory of Eco-Textiles, Ministry of Education, Jiangnan University*

1800 Lihu Avenue, Wuxi 214122, China huiqiao@jiangnan.edu.cn

[d]*Department of Chemistry and Applied Biological Sciences, South Dakota School of Mines and Technology, Rapid City, SD, 57701*

[e]*Construction & Operations Management, South Dakota State University, Brookings, SD, 57007, USA.*

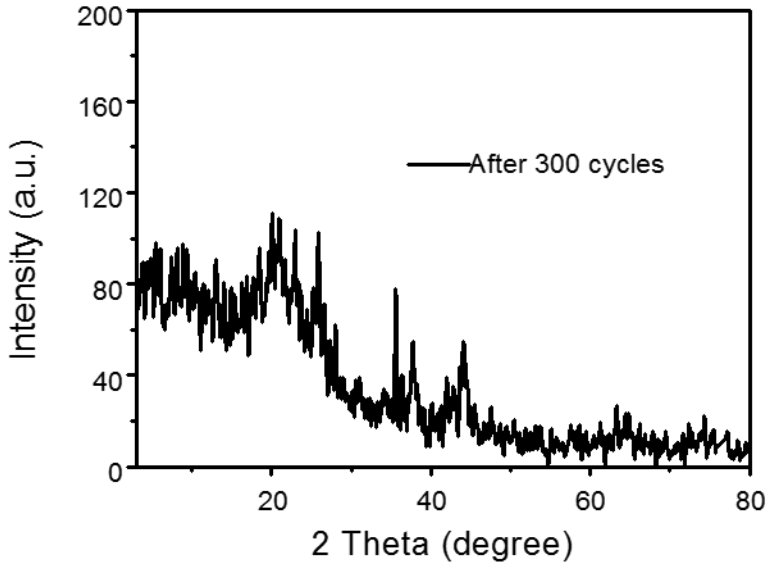

Figure S1. XRD characteristic of the ECF electrode after 300 cycles at 0.1 A/g.

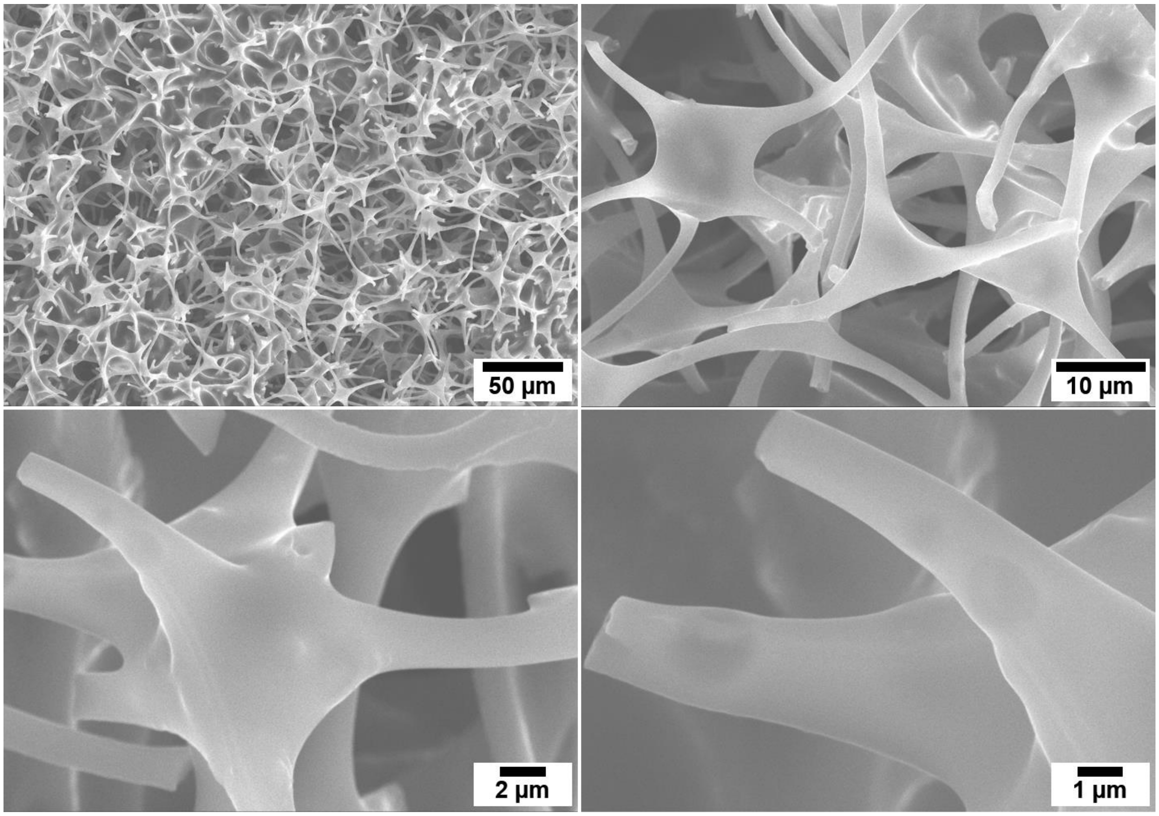

Figure S2. SEM images of the ECF electrode after 100 cycles at 0.1 A/g.

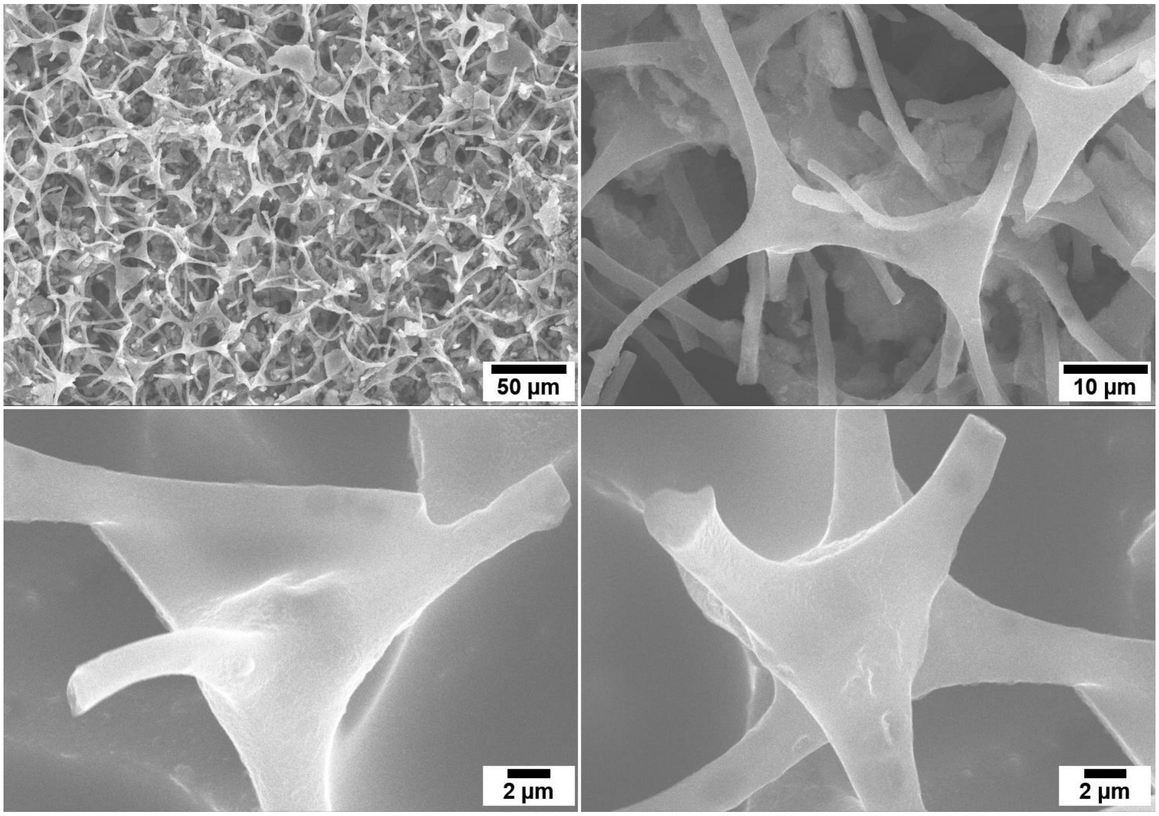

Figure S3. SEM images of the ECF electrode after 300 cycles at 0.1 A/g.
